# An overview of preclinical models of traumatic brain injury (TBI): relevance to pathophysiological mechanisms

**DOI:** 10.3389/fncel.2024.1371213

**Published:** 2024-04-12

**Authors:** Arman Fesharaki-Zadeh, Dibyadeep Datta

**Affiliations:** ^1^Department of Neurology and Psychiatry, Yale University School of Medicine, New Haven, CT, United States; ^2^Division of Aging and Geriatric Psychiatry, Alzheimer’s Disease Research Unit, Department of Psychiatry, New Haven, CT, United States

**Keywords:** traumatic brain injury (TBI), chronic traumatic encephalopathy (CTE), animal models of TBI, stress, neuroinflammation, calcium dysregulation, tauopathy

## Abstract

**Background:**

Traumatic brain injury (TBI) is a major cause of morbidity and mortality, affecting millions annually worldwide. Although the majority of TBI patients return to premorbid baseline, a subset of patient can develop persistent and often debilitating neurocognitive and behavioral changes. The etiology of TBI within the clinical setting is inherently heterogenous, ranging from sport related injuries, fall related injuries and motor vehicle accidents in the civilian setting, to blast injuries in the military setting.

**Objective:**

Animal models of TBI, offer the distinct advantage of controlling for injury modality, duration and severity. Furthermore, preclinical models of TBI have provided the necessary temporal opportunity to study the chronic neuropathological sequelae of TBI, including neurodegenerative sequelae such as tauopathy and neuroinflammation within the finite experimental timeline. Despite the high prevalence of TBI, there are currently no disease modifying regimen for TBI, and the current clinical treatments remain largely symptom based. The preclinical models have provided the necessary biological substrate to examine the disease modifying effect of various pharmacological agents and have imperative translational value.

**Methods:**

The current review will include a comprehensive survey of well-established preclinical models, including classic preclinical models including weight drop, blast injury, fluid percussion injury, controlled cortical impact injury, as well as more novel injury models including closed-head impact model of engineered rotational acceleration (CHIMERA) models and closed-head projectile concussive impact model (PCI). In addition to rodent preclinical models, the review will include an overview of other species including large animal models and *Drosophila*.

**Results:**

There are major neuropathological perturbations post TBI captured in various preclinical models, which include neuroinflammation, calcium dysregulation, tauopathy, mitochondrial dysfunction and oxidative stress, axonopathy, as well as glymphatic system disruption.

**Conclusion:**

The preclinical models of TBI continue to offer valuable translational insight, as well as essential neurobiological basis to examine specific disease modifying therapeutic regimen.

## 1 Introduction

**FIGURE 1:**
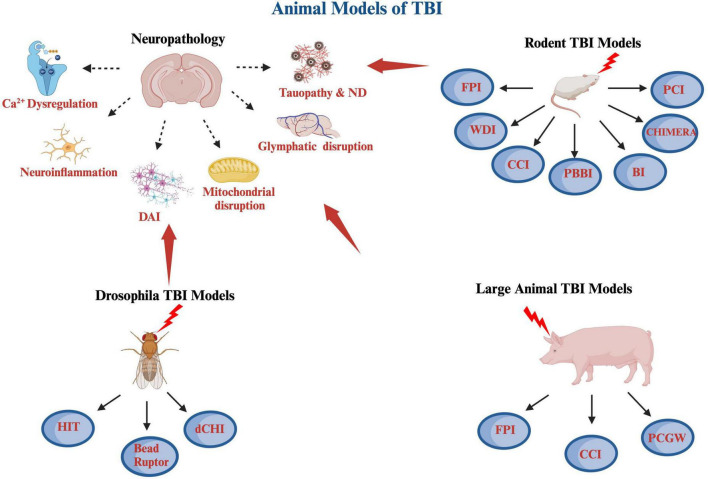
A schematic diagram of various animal models of TBI covered including rodent, large animals and *Drosophila*, as well as some of the pathological hallmarks of TBI. The rodents TBI models include fluid percussion injury (FPI), weight drop injury (WDI), controlled cortical impact (CCI), penetrating ballistic-like brain injury (PBBI), blast injury (BI), closed-head impact model of engineered rotational acceleration (CHIMERA), and closed-head projectile concussive impact model (PCI) models. The large animal TBI models include fluid percussion injury (FPI), controlled cortical impact (CCI), and penetrating craniocerebral gunshot wounds (PCGW) models. The *Drosophila* models include high impact trauma (HIT), Omni Bead Ruptor-24 Homogenizer model, and *Drosophila* closed head injury (dCHI) models. Also included are some of the pathological hallmarks of TBI, which are calcium dysregulation, neuroinflammation, diffuse axonal injury (DAI), mitochondrial dysregulation and oxidative stress, glymphatic system disruption as well as tauopathy and neurodegeneration (ND). This diagram was produced using Biorender.com.

Traumatic brain injury (TBI) is a significant public health problem, as there are approximately 1,600,000–3,800,000 concussions/mild TBI being reported annually in the United States (Peterson et al., 2019; Sanchez et al., 2021). The vast majority of TBI cases are concussions/mild TBIs (mTBIs) (Langlois et al., 2006). The remaining TBI subtypes include moderate and severe TBIs, resulting in significant morbidity and mortality and accounting for one in every three deaths due to injury (Abdelmalik et al., 2019). The majority of mTBI patients recover within 10–14 days, but as per results of the recent Transforming Research and Clinical Knowledge in TBI (TRACK-TBI) study, 13.5% of participants with mTBI experience poor cognitive outcome and persistent postconcussive symptoms (Schneider et al., 2022). TBI is a highly heterogeneous medical condition, with widely variant mechanism of injury, including falls, motor vehicle accidents, sport related concussions, as well as assaults and blast injuries in a combat setting (Blennow et al., 2016). TBI involves a complex structural and neuropathological cascade, and is mechanistically conceptualized to occur in a primary and secondary injury phase (Davis, 2000; Xiong et al., 2013). The primary injury is the result of direct mechanical impact involving structural deficits and leading to contusions, blood vessel damage and subsequent hemorrhage, as well as axonal shearing injuries (Gaetz, 2004; Cernak, 2005). The secondary injury, which could start within minutes post-injury, a delayed complex process leading to chronic neuropathological changes, including metabolic perturbation, neuroinflammation, oxidative stress, diffuse axonal injuries, neurovascular changes, and could ultimately involve neurodegenerative process, depending on the severity of injury (Greve and Zink, 2009; Cornelius et al., 2013). The clinical TBI cases are inherently heterogenous due to variation of location, nature and severity of the primary injury, as well as age, premorbid medical conditions, genetic background including APOE4, as well as injury specific parameters (Covington and Duff, 2021). Animal models of TBI offers a number of distinct advantages, which include relatively homogenous mechanism of injury, uniformity in age, sex and genetic background, as well as predetermined injury parameters corresponding to the designated injury severity (Xiong et al., 2013). Given the relatively limited animal life span, one can examine the chronic neuropathological sequelae, as well as the neurobehavioral deficits. Furthermore, animal models provide a unique biological substrate to study potential disease modifying therapeutic regimen for chronic neurobehavioral and neuropathological sequelae of TBI (Tang et al., 2020). The current review provides a comprehensive overview of animal TBI models including rodent models, as well as *Drosophila* and large animal models. The rodent animal models included in this study are the fluid percussion injury (FPI) model (Xiong et al., 2013), weight drop injury (WDI) model (Feeney et al., 1981), controlled cortical impact (CCI) model (Dixon et al., 1991), penetrating ballistic-like brain injury (PBBI) model (Williams et al., 2005), blast injury (BI) model (Cernak et al., 2011), as well as more novel rodent injury models, including closed-head impact model of engineered rotational acceleration (CHIMERA) model (Namjoshi et al., 2014), and closed-head projectile concussive impact (PCI) model (Leung et al., 2014). The *Drosophila* models include the high impact trauma (HIT) model (Katzenberger et al., 2013), Barekat’s Bead Ruptor Homogenizer model (Barekat et al., 2016), and the *Drosophila* closed head injury (CHI) models (Alphen et al., 2018). Also included in this review, is a brief overview of large animal models, including ferrets (Lighthall, 1988), pigs (Duhaime et al., 2000), swine (Duhaime, 2006), primates (King et al., 2010), as well as sheep (Dutchke et al., 2016). In addition, there is a brief overview of combined model of injury of TBI and chronic stress model, which have real clinical relevance in both civilian and military population (Fesharaki-Zadeh et al., 2020). There is a final overarching discussion on the key neuropathological changes post TBI, which include neuroinflammation, calcium dysregulation, DAI, as well as glymphatic system perturbations (Figure 1).

## 2 Animal models of TBI

There are currently 4 cardinal animal preclinical models of TBI used, which include fluid percussion injury model (FPI), weight drop injury model (WDI), controlled cortical impact injury (CCI), and blast/diffuse brain injury model (Chiu et al., 2016; Younger et al., 2019; Figure 1).

### 2.1 Fluid percussion injury model (FPI) model

In the FPI model, animals undergo craniotomy to expose a portion of the dura, prior to injury being conducted using a fluid pulse (Xiong et al., 2013). The FPI model has been established as one of the most widely used methods of inducing injury and has been used in a variety of animal species including rabbit (Härtl et al., 1997), dog and sheep (Millen et al., 1985), cat (Povlishock et al., 1983), pig (Armstead and Kurth, 1994), and especially rodents including mouse (Carbonell et al., 1998) and rats (Thompson et al., 2005). The injury involves a locally diffuse injury involving cortical contusion and diffuse subcortical neuronal injury, induced by a pendulum striking a piston at the end of a saline-filled cannula (Ma et al., 2019). The exposed dura of brain is subsequently impacted via a rapidly accelerating rod, with preset depth of impact. The rod is controlled by a pneumatic piston or an electromagnetic actuator (Dixon et al., 1991). Depending the location of injury relative to sagittal suture, FPI could be further subdivided into lateral FPI (LFPI) vs. central FPI (CFPI) (Xiong et al., 2013). The LPFI has the advantage of contrasting the extent of neuronal injury in the ipsilateral (injured site) and contralateral (non-injured) side of the brain (Hicks et al., 1996).

The injury typically leads to direct damage to the blood brain barrier (BBB), cortical tissue loss, acute hematoma and neuronal injury (Ma et al., 2019). Focal TBI typically leads to primary axotomy along with ischemic and destructive neuronal changes, while diffuse TBI results in diffuse axonal injury, referred to as traumatic axonal injury (TAI) in the preclinical TBI model setting. Indeed TAI can occur in the absence of focal contusion or hemorrhage (Kelley et al., 2007). The pathophysiological alterations in FPI are correlated with the pressure transients, in which upper mild, moderate or severe types of pressure impulse leads to focal and diffuse injury, whereas the lower threshold of mild pressure impulse results in diffuse injury (O’Connor et al., 2011). A mild TBI is typically defined at the pressure range of 0.9–1.5 atm (13–22 psi) (Griesbach et al., 2009; Shultz et al., 2011), moderate TBI is at 1.6–2.5 atm (23–37 psi) (Li et al., 2015; Chitturi et al., 2018), and severe injury involving pressure above 2.5 atm (37 psi) (D’Ambrosio et al., 2004; Vitarbo et al., 2004).

The FPI models have been utilized to simulate clinical TBI, without skull fracture, and involving edema, hemorrhage, and cortical gray matter changes (Thompson et al., 2005). The primary injury in FPI models involves contusion, shearing/stretching of cortical tissue, subdural hematoma and hemorrhage (Alder et al., 2011). The secondary injury mechanism involves activation of inflammatory glial cells and neuronal cell death, or neurodegenerative changes that can start as early as seconds post-injury at the ipsilateral side of the injury, and occurring mostly at the cortical sites of injury, hippocampus, thalamus, striatum and amygdala (Liu et al., 2010; Ma et al., 2019).

As stated prior, FPI models provide the capacity to induced mild, moderate or severe TBI injuries (Kinoshita et al., 2002; Ma et al., 2019). The suppression time of reflexes such as pinna, cornea, and righting has been established as a marker of neurological assessment in animal model immediately post FPI (Schmidt and Grady, 1993). The righting reflex is commonly used an indicator of injury severity in animal models. The neurobehavioral domains that are commonly assessed in FPI include the functional outcomes of motor, cognition, and depression or anxiety-like behaviors. The injury induced motor deficits in rodents have been assessed by beam balance, beam walking test, ladder rung walk test, as well as inclined plane test, rotating pole test, rope grip, and rota-rod test (Hicks et al., 1996; Alessandri et al., 2002; Clough et al., 2007; Gold et al., 2013). In FPI models, the location of craniotomy plays a key role in determining the extent of cognitive deficits. A small shift of 1–2 mm craniotomy location from the midline FPI can significantly alter the cognitive outcome, as such midline FPI has been reported to produce more severe cognitive impairments than the LFPI (Vink et al., 2001; Ma et al., 2019). Cognitive deficits in FPI models have been widely reported in rodent blunt TBI models, based on performance on Morris water maze (MWM) (Deng-Bryant et al., 2016). Anxiety like symptoms have been reported in mild LFPI injured animals, as measured by increase in plus maze time spent in open arms at 24 h post-injury compared to sham controls, with diminishing effect at 4 weeks post-injury (Shultz et al., 2011). Depression like phenotype has also been reported in moderate LFPI models, as measured by novelty suppressed feeding, forced swim, and social interaction test (Kuo et al., 2013).

One of the potential limitations of the use of the FPI model, is the current lack of standardized parameters, including peak pressures, and duration of injuries across laboratories, and introduce an inherent variability in the injury outcome measures (Lyeth, 2016). There are also intrinsic challenges and complications including the potential for infection, given the requirement for craniectomy using the FPI model (Lifshitz et al., 2016).

### 2.2 Weight drop injury model (WDI)

The WDI model utilizes the gravitational forces of a free-falling weight to induce focal or diffuse brain injury (Feeney et al., 1981; Ma et al., 2019). There are a number of distinct WDI models, which include Feeney’s WDI, Shohami WDI, Shapira WDI and Marmarou WDI (Shapira et al., 1988; Foda and Marmarou, 1994). There are two distinct ways to vary the severity of injury, which includes altering the weight and height of the object used in WDI (Kalish and Whalen, 2016).

Feeney’s WDI involves the delivery of impact to the intact dura, via a craniotomy resulting in cortical contusions, hemorrhagic lesions, BBB damage, infiltration of immune cells and activation of glial cells (Dail et al., 1981; Feeney et al., 1981; Uhl et al., 1994; Bellander et al., 1996; Mikawa et al., 1996; Holmin et al., 1997; Morales et al., 2005). In the Feeney’s model, injuries generally follow a specific pattern, commencing with hemorrhaging in the white matter region near the injury site, progressing to the formation of a necrotic cavity within 24 h, and subsequently expanding and evolving over the following 2 weeks (Dail et al., 1981; Feeney et al., 1981; Xiong et al., 2013). Past studies involving rats have documented enduring deficits that persist for more than 90 days (Feeney et al., 1981; Gasparovic et al., 2001).

The Shohami WDI model is a closed head injury (CHI), which is induced using a weight-drop impact on one side of the unprotected skull (Shapira et al., 1988; Shohami et al., 1988). This injury leads to blood-barrier disruption, cerebral edema, persistent neurological deficits, and neuronal loss (Kalish and Whalen, 2016). A neurological severity score (NSS), has been utilized to assess for motor and cognitive deficits post-injury (Shapira et al., 1993). The NSS score closely approximates the degree of neuropathological severity (Shapira et al., 1993).

A closed head WDI model was developed by Shapira et al. (1988), which was further modified by Chen et al. (1996), by fixing of head of the animal on a hard platform for further modification of acceleration diffuse injury (Shapira et al., 1988; Chen et al., 1996).

The Marmarou WDI is distinct from Shohami, Feeny, or Shapira’s models, as it simulates diffuse acceleration injury, representing a diffuse brain injury seen in falls or car accidents (Marmarou et al., 1994), resulting in mild to severe hemorrhage, cell loss, diffuse axonal injury, and astrogliosis (Albert-Weissenberger et al., 2012). The Marmarou model was designed to simulate motor vehicle accidental injuries caused by rotational injuries following linear acceleration injuries ultimately leading to diffuse/traumatic axonal injury (TAI) (Wang et al., 2010). In this model, death may result from potential respiratory depression, and the utilization of mechanical ventilation has proven to be an effective method for lowering post-injury mortality rates (Foda and Marmarou, 1994; Marmarou et al., 1994).

The Wayne state model, developed by Kane et al. (2012), uses a 95 g weight dropped from a height of 1 m onto the head of an anesthetized mouse, in a suspended position on an aluminum foil (Kane et al., 2012). The authors examined mice after repeated injuries, and reported reproducible cognitive deficits due to injury (Kalish and Whalen, 2016). There was reported active gliosis, and elevated tau phosphorylation, in the absence of significant microglial activation, β-amyloid deposition, BBB compromise, or cortical white matter loss (Kane et al., 2012).

A permutation of Marmarous WDI model is the Maryland model, which involves frontal acceleration, as well as lateral impact on a helmet-protected head to mimic football related injuries (Kilbourne et al., 2009; Viano et al., 2009; Ma et al., 2019). WDI injury severity, though less systematically studied, is correlated with weight and height (Ma et al., 2019). To assess the degree of neurological impairment, the NSS is frequently employed (Flierl et al., 2009; Yarnell et al., 2016). The NSS is designed to assess for neurological impairment, alertness, and seeking behavior, and its score is highly correlated with severity of brain injury (Xiong et al., 2013). The WDI model leads to learning and memory deficits, as measured by passive avoidance, Y/T maze, radial water maze, novel object recognition, fear conditioning, MWM, and Barnes maze. For affective behavior testing, elevated plus maze or zero maze and social testing have been commonly utilized, with mixed outcome (Bodnar et al., 2019).

The WDI models effectively simulate focal or diffuse TBIs and provides the capacity to induce graded DAIs. Given the potential for rebound impacts and variations in impact velocity, the WDI model inherently exhibits a certain degree of heterogeneity (Ma et al., 2019). The major advantage of the WDI model is its ability to provide a simple and relatively inexpensive way to reproduce graded DAI. However, there is also a risk of rebound impact and variation of impact velocity, in turn limiting the accuracy of injury severity (Ma et al., 2019). There are inherent limitations to the WDI model, which includes variations in direct vs. indirect impact to the skull, impact location, mobility of the head, the surface on which the animal is positioned, as well as projectile shape and material (Bodnar et al., 2019).

### 2.3 Controlled cortical impact model (CCI)

In the CCI model, the brain is impacted via surgically exposed dura by a rapidly accelerated rod with preset computer guided depth of impact (Ma et al., 2019). The rod comes in different size and shape variation depending on species involved, and is manipulated by a pneumatic piston or an electromagnetic actuator (Dixon et al., 1991). The impact leads to blood-brain barrier damage, cortical encephalomalacia, and subdural hematoma. The distinct advantage of the CCI model is the ease of which mechanical factors, including time, velocity and depth of impact, can be manipulated (Mao et al., 2006; Wang and Ma, 2010). The CCI model leads to significant cortical contusion and neurodegenerative changes within the ipsilateral cortical site. The CCI model has also been modified into closed skull injury, as well as repetitive mild TBI model (rmTBI) (Smith and Hall, 1996; Tang et al., 2020). The newer iterations of the CCI model, has expanded our collective understanding of cellular, biochemical, and molecular mechanisms of brain injury, by controlling parameters such as depth and velocity of impact (Osier and Dixon, 2016; Fesharaki-Zadeh et al., 2020).

The CCI model utilizes the stereotaxic apparatus and electromagnetic actuator to simulate a range of immune-histological and behavioral outcomes due to mild, moderate and severe TBI injuries. Depth and velocity of the impactor are the cardinal parameters determining the severity of injury in the CCI model (Yu et al., 2009; Washington et al., 2012). The depth of injury is an especially robust parameter to manipulate the severity of injury, ranging from mild to severe. Washington et al. (2012) simulated brain injury at depth of 1.5 mm, 2.0 mm, and 2.5 mm and a preset velocity of 5.25 m/s, to produce a model of mild, moderate and severe TBI. A similar study by Wang et al. (2016), used different velocity (velocity of 3.5 m/s) and depth (0.2, 1.0, and 1.2 mm, respectively) parameters to induce mild, moderate and severe TBI. The neurological deficits in CCI injury in rodents, including deficits in spatial memory tasks such as MWM, is highly correlated with severity of injury (Marklund and Hillered, 2011). Neurocognitive deficits post CCI injury has also been shown to be persistent up to one year post-injury due to great heterogeneity of proposed CCI models, there are recent efforts to standardize the injury parameters (Siebold et al., 2018). Another distinct advantage of the CCI model, is a lack of risk of rebound injuries (Xiong et al., 2013).

### 2.4 Penetrating ballistic-like brain injury model

Penetrating ballistic-like brain injury (PBBI) is induced by transmission of projectiles with accompanying high energy and shock waves, leading to formation of temporary cavity in the brain (Williams et al., 2005). The injury sequelae is dependent on the specific anatomical path of the projectile, as well as the corresponding energy discharge (Williams et al., 2006a,b). Experimental PBBI simulate gunshot wounds, and have been conducted in cats, as well as rodents (Carey et al., 1989, 1990; Davis et al., 2010).

Penetrating ballistic-like brain injury (PBBI) injury leads to significant gray and white matter damage, brain edema, seizures, cortical spreading depression and accompanying neuroinflammation, and subsequent neurocognitive and sensorimotor impairment (Williams et al., 2007). There are more novel, non-fatal variation of PBBI model that involve a modified air rifle using a pellet (Plantman et al., 2012). This injury model leads to cavity formation, white matter degeneration, hemorrhage, edema and accompanying gliosis. To simulate the ballistic effect of the injury, a PBBI rat model has been developed to represent immediate and subacute changes in intracranial pressure (ICP) (Wei et al., 2010), as well as BBB permeability changes and brain edema. Persistent neurofunctional changes include persistent motor deficits, as measured by performance on balance beam and rotarod task, and cognitive changes, reflected by spatial learning impairment in MWM task (Shear et al., 2010, 2011), which are correlated with injury severity. Similar to other animal brain injury models, the PBBI model leads to parenchymal edema, increased ICP, white matter injury and persistent inflammation (Williams et al., 2006a,^2007^). PBBI has also been shown to lead to activation of inflammasome, based on expressions of IL-1β, and IL-18 (Lee et al., 2018). Distinctly, PBBI model leads to extensive intracerebral hemorrhage due to penetrating nature of injury. The PBBI model has the unique capacity to capture the temporal ballistic brain injury, and can effectively simulate moderate to severe brain trauma in a combat setting (Xiong et al., 2013).

### 2.5 Blast injury model

Many military servicemen have experienced blast injuries, without overt TBI (Warden, 2006; Benzinger et al., 2009). Blast-induced traumatic brain injury (bTBI) has been called the “signature wound” of the Afghanistan and Iraq conflicts. While it has been mostly a significant military based health issue, it can certainly be a civilian health threat (Hicks et al., 2010). There are many iterations of bast wave animal models, mostly involving rodents (Bauman et al., 2009; de Lanerolle et al., 2011). The blast injury utilizes a compression-driven shock tube, and animals are placed inside a Kevlar thoracic protective vest, which in turn encases the thorax and part of the abdomen and significantly reduces air blast mortality, and diminished axonal fiber degeneration (Long et al., 2009). The most basic form of blast wave has been described as Friedlander waveform in the driven chamber, quantified by a pressure sensor, and can vary based on distance from membrane, as well as angle of the tubing (Cernak et al., 2011; Wang et al., 2016). Blast injury has a distinct pathophysiological pattern, which include diffuse cerebral edema, extreme hyperemia, delayed vasospasm, as well as DAI, as reported in rats studies (Garman et al., 2011).

Prior studies using low intensity blast injury, have also reported increases in ICP and persistent cognitive deficits, including social recognition, spatial memory and motor coordination (Säljö et al., 2009). Blast-exposed mice have been reported to display phosphorylated tauopathy, myelinated axonopathy, microvasculopathy, persistent and chronic neuroinflammation in the absence of overt tissue damage and hemorrhage (Goldstein). Head immobilization also plays a major role, as it has been shown to prevent blast injury-induced memory and neurobehavioral deficits (Bhattacharjee, 2008). Clinically, blast injuries have been described previously as “shell shock.” The survivors of the blast injury presented with symptoms of insomnia, vertigo, and persistent memory deficits, with 36% of the group displaying abnormal electroencephalogram (EEG) signals (Cernak et al., 1999). Blast injuries are proposed to involve transferred kinetic energy, leading to low-frequency stress waves, involving rapid physical movement, in turn leading to displacement and deformation of the medium (Cernak, 2015).

Given the prevalence and nature of mTBI in a combat setting, there is an urgent need to explore the underlying pathophysiology of low-intensity blast (LIB)-induced brain injury. The LIB model typically utilizes exposure to magnitude 46.6 kPA and a maximum of 8.7 PSI (aka 60 kPA)/ms blast, as opposed to > 100 kPA in the case of moderate to high intensity blast (Song et al., 2018a,^2019^). Despite the lack of mortality or gross macroscopic injuries, there are measurable neurobehavioral deficits, diminished mitochondrial fission-fusion activities, bioenergetic failure, increased oxidative stress, diminished levels of mitophagy, acute increase in compensatory respiratory activity, as well as axonal myeline injury (Song et al., 2018c). The myelinated axonal injury has been more pronounced in the subacute 7 days post-injury (DPI), as opposed to the chronic 30 DPI (Song et al., 2018b). A single LIB exposure has been shown to lead to increased levels of total tau, p-tau, and/or Aβ. Furthermore, the density of asymmetrical synapses was significantly diminished in the cortex of blast animals. Synaptic loss could be the secondary effect of diffuse axonal injury, and as shown in a rat model of CCI (Gao et al., 2011), it could be more of an initial acute/subacute response, as per prior studies (Scheff et al., 2005).

### 2.6 Closed-head impact model of engineered rotational acceleration (CHIMERA) model

Closed-head impact model of engineered rotational acceleration (CHIMERA) is a relatively novel model of TBI. The model was developed to simulate the majority of clinical mild TBI cases (Namjoshi et al., 2014). CHIMERA model is uniquely designed to integrate biomechanical, behavioral and neuropathological analyses using a preset defined energy with unconstrained head motion. CHIMERA model has shown excellent reproducibility using two impacts separated by 24 h, as captured by comparable repeated measures of head trajectory, linear velocity and acceleration, head displacement, angular velocity and acceleration and angle of impact on two consecutive days. A major limitation of CHI and CCI models is that the injury parameters including mechanical loading, method used for mechanical loading, and animal’s head response to injury are often not well-controlled, and these factors in turn lead to considerable experimental variation in neurobehavioral, and immunohistological outcomes. Repeated CHIMERA based injuries was reported to lead to prolonged loss of righting reflex, neurocognitive and motor deficits, as well as anxiety-like behavior. The repeated CHIMERA brains showed measurable inflammatory response based on heightened IL1β and TNFα signals, as well as enhanced tau phosphorylation (Namjoshi et al., 2014). Namjoshi et al. (2014, 2017), reported CHIMERA induced increases in righting time, neurological severity, as well as persistent motor deficits and anxiety like behavior. Furthermore, the authors reported post-TBI persistent microgliosis, based on heightened Iba1 levels, in several major white matter tracts including the optic tract, olfactory nerve layer, corpus callosum, and brachium of superior colliculus. There was also a reported significant astrocytic activation in the corpus callosal area, based on enhanced GFAP signal. There were also significant energy dose-dependent increases in axonal damage in the major white tracts based on increased silver staining update, which demonstrated a persistent increase over the 14 days following the injury within the corpus callosal tracts. However, there were no reported significant changes in total tau, phosphorylated tau, or the ratio of phosphorylated tau:total tau (Namjoshi et al., 2017).

Closed-head impact model of engineered rotational acceleration (CHIMERA) model has offered versatility in terms of controlled severity of injury, ranging from 0.7 joules simulating a mild TBI model to 2.5 joules simulating a more severe injury types, based on robust acute neurological deficits, enhanced plasma total tau and neurofilament-light levels, increased pro-inflammatory markers, microgliosis and BBB compromise (Bashir et al., 2020). Repeated CHIMERA model has been shown to be a useful translational model for chronic neurobehavioral sequelae of rmTBI, including profound inhibition of extinction of fear memories, consistent with features of posttraumatic stress disorder (PTSD), as well as chronic microgliosis, axonal injury and astrogliosis (Cheng et al., 2019). Analogous repetitive CHIMERA model in rats has been reported to result in impulsivity using delayed discounting task, as well as neuropathological changes comprised of white matter inflammation, tau immunoreactivity and degeneration involving the optic tract and corpus callosum, and prominent gray matter gliosis in the olfactory tubercle (Vonder Haar et al., 2019). A higher intensity CHIMERA model, “modCHIMERA,” using two injury severity levels 1.7 and 2.1 joules, resulted in persistent neurobehavioral deficits, including decreased spontaneous behavior, spatial learning and memory deficits, and socialization at 1 month, as well as significant microglial activation in cortical and subcortical areas including hippocampus and lateral septal nucleus (LSN) and significant axonal injury manifested by increased β-APP accumulation in the major white matter tracts including corpus callosum (CC), anterior commissure (AC), hippocampal commissure (HC), and fimbria (Sauerbeck et al., 2018). CHIMERA model has also been more recently applied to gyrencephalic ferrets, also reporting axonal injury based on enhanced APP signal, and neurofilament M (RMO) levels, as well as increase in tau phosphorylation AT180 at the base of the sulci by using a higher intensity impact, in turn offering a possible CTE animal simulation (Krieg et al., 2023).

### 2.7 Closed-head projectile concussive impact (PCI)

The projectile concussive impact (PCI) model was developed at Walter Reed Army Institute of Research (WRAIR), which simulates a closed-head impact (Chen et al., 2012; Leung et al., 2014; Michalovicz et al., 2023). The PCI device is comprised of an elevated platform, which can be adjusted to different heights, and situated above a heating unit. In order to launch a projectile, microcentrifuge tubes filled with dry ice and tightly sealed. The capped tubes are vertically inserted into the heating unit. Upon applying heat to the microcentrifuge tubes, it triggers a rapid sublimation of the dry ice, resulting in a mounting of internal pressure, in turn forcing the cap to burst off the tube and launch as an intact projectile (Chen et al., 2012). The severity of PCI model can be adjusted based on the material and mass of the ball-bearing projectile. One of the primary advantages of the PCI model is the ability to inherent ability to control, reproduce and quantify the mechanical forces used to induce the injury. Additional distinct advantages of the PCI model include high throughput capacity, relatively basic design and ease of fabrication (Leung et al., 2014). Rodents undergoing PCI injury, have shown to have more neurobehavioral deficits using a revised neurobehavioral severity scale (NSS-R), as well as delayed righting reflex, up to 30 min depending on the severity of the injury (Leung et al., 2014). Depending of the severity of injury (mild vs. severe TBI), varying degree of neuroinflammation can be induced, with the severe TBI (sTBI) inducing a significant rise in inflammatory cytokine mRNAs in multiple cortical regions including the hippocampi and cerebellum within 6 h of injury, along with pronounced gliosis. In addition, elevated level of cortisol, vascular endothelial growth factor (VEGF), indicative of a weakened blood brain barrier, as well as decreased level of acetylcholine has been reported. The mTBI model was shown to have an intermediate level of neuroinflammatory state (Michalovicz et al., 2023). Due to likely combined linear and rotational injury, there are reported acute increases in ß-APP staining, indicative of tissue deformation and DAI (Leung et al., 2014).

Although the majority of animal TBI models have focused on rodent models, other animal models provide their own unique vantage points, as described in the following section. Additional TBI models include *Drosophila*, as well as large animal models as described. The *Drosophila* model offers the advantage of studying a complex phenomenon in relatively narrow time span, whereas the large animal models offer a potentially closer proximation to clinical TBI studies. The following section offers a brief overview of these major TBI models.

## 3 *Drosophila* models of TBI

*Drosophila melanogaster* fly model provides a unique substrate to study TBI. The fly genome is comprised of 13500 genes, with approximately 70% of genes also recognized in human brain diseases (Jeibmann and Paulus, 2009). The *Drosophila* brain is quite similar with that of mammals, with analogous neuronal population groups and neurotransmitters (McGurk et al., 2015), in turn providing the necessary biological substrate to study neurodegenerative diseases such as Huntington’s disease, motor neuron disease, as well as Parkinson’s disease and Alzheimer’s disease (AD) (Moloney et al., 2010). There are a number of notable advantages that the fly TBI model would provide, which include the ability to use a large number of animals, which can be both rapidly and relatively inexpensively examined to assess for correlations between injures and outcomes, studying the underlying molecular and genetic pathways that are activated post-injury, as well as studying the relevant outcomes over the entire lifespan of the flies (Katzenberger et al., 2013). The fly brain is organized into three distinct regions, including the protocerebrum, deutocerebrum and tritocerebrum, which are analogous regions to forebrain, midbrain and hindbrain in humans (Reichert, 2005).

Katzenberger et al. have developed a “high-impact trauma” (HIT) device, which is comprised of a metal spring attached at one end to a wooden board, with the free end position over a polyurethane pad. There is a standard plastic vial, which contains unanesthetized flies, which are position at the bottom of the vial by the aid of a stationary cotton ball, in tun connected to the free end of the spring. Once the spring is deflected and released, the vial would impact the polyurethane pad, resulting in delivery of a mechanical force as the flies contact the vial and rebound (Katzenberger et al., 2013). There is an inherent lack of penetrating injuries and randomness of the injured region, which also simulate a closed-head TBI in human population. The HIT device can also be adjusted by varying the extent of the spring deflection or number of strikes. Compared to uninjured flies, flies that received one strike had a significantly reduced median as well as maximum lifespan, with additional strikes leading to further diminution of median and maximum lifespan, in turn suggesting subthreshold injuries can indeed lead to long term secondary injury consequences. The authors also reported significantly worse neurodegenerative outcomes in older flies, manifested by appearance of vacuolar lesions in the brain neuropil region. There were no discernible mortality difference between the male and female flies (Katzenberger et al., 2013). These findings are concordant with the previously reported lack of difference of long term mortality outcome between male and female TBI patients (Coimbra et al., 2003).

In addition to the HIT model, Barekat et al. (2016) introduced an alternative model of *Drosophila* TBI using the Omni Bead Ruptor-24 Homogenizer platform. The injury parameters used included intensity [meters/second, (m/s)], duration (s), and number of injury bouts. The Bead Ruptor Homogenizer model was also shown to induce altered sleep/awakening cycle-related behavior, concordant to what has been reported in the clinical population (Barekat et al., 2016).

An additional *Drosophila* model was designed by Allada lab, using *Drosophila* closed head injury (dCHI) (Alphen et al., 2018). The dCHI involves delivery of preset, non-penetrating strikes to the heads of un-anesthetized flies, using the forward movement of a brass block. The *Drosophila* to be injured is immobilized and placed in a modified 200 ml pipette in front of the block. The dCHI model induces analogous TBI phenotype, including increased motor deficits, neuronal cell death, increased mortality, and altered sleep/wake cycle (Alphen et al., 2018). A modified dTBI model involves using a piezoelectric actuator that rapidly compresses the head of *Drosophila* with precision (Saikumar et al., 2020). dTBI caused dose dependent and long-lasting neurological deficits, including deficits in righting reflex, climbing, and diminished life span. There was associated neurohistological characteristic hallmarks of injury including disruption of the glial process, oxidative stress, as well as increased brain vacuolization (Saikumar et al., 2020).

## 4 Large animal models of TBI

Rodent TBI models cannot adequately address the appropriate modeling for biomechanical and physiological parameters of injury, given the fact that rodents have largely lissencephalic brains, whereas humans have gyrencephalic brains. The presence of gyri can significantly affect the movement of the brain within the skull, and lead to more brain deformation (Finnie, 2001; Vink, 2018). The maximum mechanical stress in brain injuries is experienced far away from the surface of the brain, and at the depth of the sulci, which is also concordant with the neuropathological description of chronic traumatic encephalopathy (CTE), defined by tau phosphorylation deposits in the peri-vascular and sulci regions (Barrio et al., 2015; McKee et al., 2015).

The majority of earlier large animal studies involved models with direct brain deformation in an attempt to replicate clinical TBI (Vink, 2018). One such model was the fluid percussion injury (FPI) used in cats (Sullivan et al., 1976), with its subsequent use in dogs, and sheep (Millen et al., 1985), swine (Solomon et al., 2011), as well as rabbits (Härtl et al., 1997) and finally rodents (Dixon et al., 1987; McIntosh et al., 1987). There was a subsequent development of an alternative model, using controlled cortical impact (CCI) utilized in ferrets (Lighthall, 1988), pigs (Duhaime et al., 2000; Manley et al., 2006), swine (Zheng et al., 2014) primates (King et al., 2010), as well as sheep (Dutchke et al., 2016). CCI models in piglets have also been utilized in order to assess for peripheral biomarkers, as well as translational interventions (Margulies et al., 2015). The primate CCI model, involved impact to the right frontal cortex, with reported neuronal loss, vascular disruption, edema and resultant inflammatory process. The non-human primate studies also provide means of assessment for fine motor and sensory outcomes, which are not available in rodent models. Blast injury was first utilized in rabbits to study cardiac and lung function post-injury (Clemedson and Hultman, 1958), prior to its use to model TBI (Cernak, 2015). Penetrating craniocerebral gunshot wounds (PCGWs) have also been modeled in cats (Carey et al., 1989, 1990), primates (Crockard et al., 1977), as well as rats (Nakao et al., 2010). Given the high mortality rate of PCGW model, an alternative bilateral frontal lobe PCGW was also established in swine, with a lowered mortality rate (Lu et al., 2015).

Rotational injury is a well-known mechanism of TBI, as previously explored (Smith et al., 1997; Smith et al., 2000). There is a well-established and clinically relevant swine model of concussion, which induces evolving axonal injury using a HYGE device, a pneumatic actuator able to convert linear motion to angular (rotational) motion (Song et al., 2022). The swine rotational injury was demonstrated to involve significant axonal pathology, characterized by swollen axonal bulbs with neurofilament accumulation (Browne et al., 2011). The HYGE Swine TBI model led to significant loss of voltage gated sodium channels (NaChs), and their associated anchoring proteins at the nodes of Ranvier (NOR). Furthermore, there were significant accumulation of amyloid precursor protein (APP) across the white matter (Song et al., 2022). The swine TBI model offers a robust translational simulation for DAI (Cullen et al., 2016; Zhan et al., 2022).

There are a few challenges involving the use of large animals for TBI studies, which include a relatively larger expense, in terms of both cost, as well as holding facilities. There is also the need for higher level of care for larger animals, given their inherent longer life span, in turn, requiring more specialized care, both before and after trauma. However, the study of TBI in large animals provide an advantageous perspective of closer cortical injury simulation to the clinical population, as well as the possibility of unique neurodevelopmental studies post-TBI (Duhaime, 2006; Pareja et al., 2016; Vink, 2018).

## 5 Combined stress and TBI models

**FIGURE 2:**
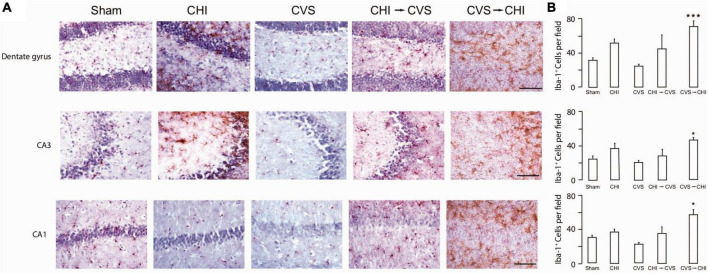
Chronic variable stress proceeding close head injury (CVS → CHI), a rodent C57BL/6J model for PTSD preceding TBI, resulted in **(A)** increased Iba-1^+^ Cell Number in Multiple Hippocampal Regions, including dentate gyrus (top panel), CA3 (middle panel) and CA1 (bottom panel). **(B)** Corresponding Iba-1^+^ Cell Numbers are shown here, indicating significantly enhanced neuroinflammatory response in dentate gyrus, CA3 and CA1 regions. Images are based on study completed by Fesharaki-Zadeh et al. (2020). *p < 0.05 and ***p < 0.0005.

Risk factors associated with worsened clinical outcomes post TBI include older age at the time of injury, limited educational background, lower socioeconomic status, as well as prior psychiatric illnesses including PTSD, depression and anxiety (Lupien et al., 2009; Nugent et al., 2011; Sanchez et al., 2021). Prior studies have also shown that TBI increases the probability of PTSD (Bryant, 2011; Yurgil et al., 2014; Spadoni et al., 2018), as well as depression (Rosenthal et al., 1998; Maller et al., 2010; Lavoie et al., 2017) and anxiety (Moore et al., 2006; Mallya et al., 2015; Osborn et al., 2016). There are a number of combined preclinical stress and TBI models that have been proposed (Kwon et al., 2011; Xing et al., 2013; Ojo et al., 2014; Sierra-Mercado et al., 2015; Davies et al., 2016; Fesharaki-Zadeh et al., 2020). Fesharaki-Zadeh et al. (2020) examined the interaction of TBI and PTSD, using the preclinical models of closed head injury and Chronic Variable Stress, respectively. The result of the study demonstrated an asymmetrical and synergistic relationship between TBI and PTSD. More specifically PTSD proceeding TBI (CVS → CHI) lead to a more severe phenotype, based on performance on various neurobehavioral assays. Furthermore, the CVS → CHI group had heightened degree of inflammation, based on Iba1 immunohistological signal in hippocampal regions, with strong degree of correlation between heightened neuroinflammation and neurobehavioral deficits (Fesharaki-Zadeh et al., 2020; Figure 2). These findings are also in parallel with the larger paradigm of the global neuroinflammatory effects of chronic stress proceeding TBI.

**FIGURE 3:**
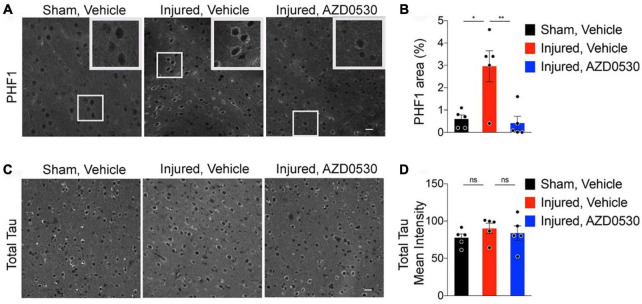
Representative images of cortical PHF-1 staining for phosphorylated-tau involving the peri-lesional, 05–1 mm medial to injury sites of 7.5 months old wild type C57BL/6J rmTBI/stress (Inj) mice treated with either vehicle vs. AZD0530 vs. **(A)**, and the corresponding quantification of PHF-1 (paired helical filaments-1) positive area **(B)**. Representative images of total Tau staining in the same three groups of mice with corresponding quantification of total Tau positive area (Panels **C,D**). As shown, AZD0530 treatment resulted in modification of tau phosphorylation in the AZD treated injured animals, with no modification effect on the total tau levels. Images are based on the study completed by Tang et al. (2020). *p = 0.0077 and **p = 0.0046.

There is a growing body of preclinical and clinical literature reporting worse neurocognitive and neurodegenerative outcomes in animals exposed to early life stress prior to TBI injury (Diaz-Chávez et al., 2020; Catale et al., 2021; Sanchez et al., 2021; Catale et al., 2022), with the clinical literature reporting mixed results (Lajud et al., 2021). The result of worsened outcome due to chronic stress could be the result of activation of HPA (hypothalamic-pituitary-adrenal), as well as SNS (sympathetic nervous system) (Nusslock and Miller, 2016), and microglial activation (Kosten et al., 2012; Nusslock and Miller, 2016; van Bodegom et al., 2017; Johnson and Kaffman, 2018). Chronic stress either in the form of stress exposure prior to TBI or in the neurodevelopmental setting, would “prime” a neuroinflammatory response prior to injury, and in turn lead to more severe neurobehavioral and neurodegenerative outcome (Nusslock and Miller, 2016; Tang et al., 2020). A recent study involved prevention of microglial/macrophage activation using GW2580 treatment, a microglial activation inhibitor without microglial ablation. The rodents treated with GW2580, had significantly improved outcome based on overall reduced microglial/macrophage activation, diminished mortality and improved functional recovery and outcome (Catale et al., 2021). These results suggest that early treatment focused on neuroinflammation, could play a key role in modifying the probability of developing neurodegenerative disorders including Alzheimer’s disease and chronic traumatic encephalopathy (CTE) (Fesharaki-Zadeh, 2023). The asymmetrical synergistic interaction of chronic stress and TBI has been utilized to create models of repetitive mild TBI and chronic stress to simulate neurodegenerative tauopathy like CTE, and examine potential disease modifying therapeutic using Fyn kinase inhibition via modulation of tau phosphorylation (Tang et al., 2020; Figure 3).

## 6 Neuropathological sequelae of TBI

Over the last few decades, the neuropathology of TBI-related degeneration is beginning to be deciphered in postmortem human tissue. Critical insights have been gleaned from rodent TBI and chronic traumatic encephalopathy (CTE) models, especially region-, circuit-, and cell-type specific molecular abnormalities that trigger the pathological sequelae. In particular, epidemiological and clinical studies have revealed shared pathological features between repeated mild TBI and Alzheimer’s disease (AD) (Blennow et al., 2016; Washington et al., 2016; Mendez, 2017), culminating in the development of dementia, including increased risk for cerebral atrophy, CTE and even Parkinson’s disease (PD). For example, analysis of large cohorts of individuals during lifetime have revealed that single mild TBI was associated with ∼20% greater risk for the development of dementia (Fann et al., 2018). These studies have reinforced the notion that TBI is not merely an acute event but has long-term ramifications that is associated with chronic disability (Wilson et al., 2017). The pathological alterations in TBI and CTE include the manifestation of hyperphosphorylation of tau leading to the formation of neurofibrillary tangles (NFTs) as a result of mitochondrial dysregulation, calcium dysfunction, and activation of inflammatory cascades, involving cortical sulci and perivascular regions in the early stages, and more global parenchymal areas in the more advanced stages (McKee et al., 2015). The following sections will highlight recent developments in the field pertinent to these areas.

### 6.1 Tau pathology

Tau is microtubule-associated protein tau (MAPT) that plays a critical role in the movement of cargo proteins throughout neurons and stabilization of microtubules, particularly within neurons. Under pathological conditions, tau is subject to several post-translational modifications (PTMs) including phosphorylation. Hyperphosphorylation of tau results in disassociation of tau from microtubules, translocation to dendrites within neurons, oligomerization and the generation of intracellular NFTs. Studies have revealed that even a single episode of TBI in ∼30% of subjects led to the NFT pathology and the onset of dementia (Johnson et al., 2012; Zanier et al., 2018). Histological examination of CTE patients with repeated trauma has revealed focal perivascular accumulation of hyperphosphorylated tau as NFTs within neurons, neuropil threads and astrocytic tangles, with a heightened propensity within the depths of cortical sulci. Clinically, patients with CTE, originally referred to as “punch-drunk syndrome” or “dementia pugilistica,” are characterized by mood and behavioral disorders and cognitive impairment (McKee et al., 2010; Omalu, 2014). The presence of phosphorylated tau lesions has been categorized as the minimum threshold requirement for CTE diagnosis. The general histopathological pattern of NFTs in CTE include several brain areas, including the neocortex, thalamus, brainstem and rarely, spinal cord. Pathology emerges focally and in discrete patches in the sulci of the neocortex, oftentimes in the superior and dorsolateral prefrontal cortex, but increasing in prevalence and spreading to temporal and parietal cortices as the degeneration progresses. With advanced stage of disease, NFTs are expressed extensively with the most intense staining pattern in the sulci with neuronal loss, accompanied by white matter pathology, glial tau pathology and gliosis, exhibiting a distinct pattern. The magnitude of tau pathology in TBI and CTE has been found to be correlated with age, duration and severity of head impact injuries, clinical signs and symptoms and survival after injury.

However, the spatial and temporal progression of NFT pathology in CTE is distinct and different from AD. In AD, tau pathology first arises in the brainstem nuclei projecting broadly to the cerebral cortex (e.g., noradrenergic locus coeruleus) and in the cerebral cortex emerges in the transentorhinal region (TRE) and then in superficial cellular layers of the entorhinal region (ER) early in the course of the illness inducing degeneration (Braak et al., 2011; Braak and Del Tredici, 2014). Tau pathology then extends to deeper layers of the ER and arises in the interconnected hippocampal formation and association cortices, sparing primary sensory and motor fields untouched until late-stage disease. Recent cryo-EM studies have revealed that the 3D conformation of tau filaments in CTE is distinctly different from AD and Pick’s disease, suggesting that the mechanisms that lead to tau trafficking and aggregation might be uniquely different in various types of dementia (Falcon et al., 2019). Furthermore, there is a general absence of tau in perivascular astrocytes in sulcal depths in AD.

Several studies in human and in rodent TBI models are beginning to unravel the phosphorylation PTM landscape, compared to other forms of dementia. The studies have revealed shared and unique p-tau epitopes on tau in patients with acute and repeated TBI. For example, histopathological studies in CTE patients have highlighted phosphorylation at Thr175, Ser422, Ser199, Ser202, Thr205 and Thr231 as important in the pathogenesis of neurodegeneration (Mufson et al., 2016; Puvenna et al., 2016; Moszczynski et al., 2018). However, future studies are needed to delineate the presence of p-tau epitopes that are emerging as fluid-based biomarkers in AD, e.g., Thr181 and Thr217, early, soluble p-tau sites that cause detachment of tau from microtubules, e.g., Ser214, and other p-tau sites that enhance tau seeding capacity, e.g., Ser262. In addition to phosphorylation, additional studies need to dissect the contribution of other PTM, such as acetylation and ubiquitination, and how they uniquely predispose alterations in the conformational state of tau to mediate propagation within afflicted brain circuits. Animal models of TBI/CTE in conjunction with postmortem human studies will be crucial for these future directions for the field.

In addition to tau pathology, there is the concurrence of other pathological phenomena in TBI/CTE, including beta-amyloid plaques, cerebral amyloid angiopathy, Lewy body disease, and TDP-43 proteinopathy which has been shown to increase in likelihood across age-span (Stein and Crary, 2020). These pathological alterations persist for months to years after acute TBI, suggesting that physical insults could trigger a pathogenic sequence of events that culminate in behavioral impairments (Goldstein et al., 2012; Johnson et al., 2012, 2013).

### 6.2 Calcium dysregulation

Calcium signaling is widely recognized as a critical determinant of synaptic plasticity, acting as a second messenger to regulate a myriad of physiological processes and intracellular signal transduction pathways. Several lines of evidence have revealed that calcium dysregulation in neurons has been hypothesized as key etiological driver of neuropathology in TBI. Calcium entry within neurons occurs through multiple sources: (1) receptor-operated calcium channels (ROCs), e.g., N-methyl-d-aspartate (NMDA) receptors, that are activated by endogenous ligand glutamate, which drives calcium influx from the extracellular space into the cytosol; (2) voltage-gated calcium channels (VGCCs) that under depolarized conditions, e.g., as a result of Na^+^ entry through ion channels or due to Ca^2+^ influx through ROCs; (3) calcium-induced calcium release from intracellular stores located on the endoplasmic reticulum (ER). This occurs through inositol 1,4,5-trisphosphate (IP_3_)-sensitive Ca^2+^ stores, and ryanodine-sensitive Ca^2+^ stores. IP_3_-sensitive Ca^2+^ stores can be activated by G protein-coupled receptors such as Group I metabotropic glutamate receptors (mGluRs) or muscarinic acetylcholine receptors, which activates phospholipase C (PLC), which in turn cleaves phosphatidylinositol biphosphate (PIP_2_), causing the release of diacylglycerol (DAG) and IP_3_. In addition to the canonical mechanisms, additional pathways include store-operated or second-messenger-operated channels (SOCs/SMOCs) that drive calcium influx when intracellular calcium levels are diminished. Another source of calcium occurs through transient receptor potential channels (TRPCs) that are located on the plasma membrane which are thought to contribute to calcium induced calcium release (CICR) in neurons. Due to the multiple routes that permit the elevation of calcium within intracellular compartments, there are several homeostatic mechanisms to constrain calcium within specific subcellular compartments. This includes calcium-binding proteins, e.g., calbindin and parvalbumin, to sequester excessive Ca^2+^ within the cytosol. There are mechanisms that also promote extrusion of Ca^2+^ from the intracellular space to the extracellular space via membrane pumps such as the plasma membrane Ca^2+^-ATPase (PMCA), and the Na^+^/Ca^2+^ exchanger which is activated by elevated levels of Ca^2+^ and is dependent on the Na^+^ gradient, regulated by the membrane Na^+^/K^+^ ATPase. Finally, intracellular organelles such as the ER, including specialized pumps such as the sarcoplasmic–endoplasmic reticulum Ca^2+^-ATPase (SERCA) on the ER membrane, and mitochondria play important roles in buffering excessive calcium, but calcium overload in these organelles can abrogate their function.

Mechanical injury from TBI can produce shearing forces that can alter the biophysical properties of calcium channels that regulate intracellular calcium influx. For example, experimental studies *in vivo* in TBI models and *in vitro* studies have revealed greater permeability of calcium-regulatory channels following mechanical injury. The upregulation of calcium conductance within neurons is likely a key mediator of secondary damage resulting in activation of signaling pathways that perturb calcium homeostasis. Consistent with this idea, acute and chronic injury in TBI is associated with activation of calcium-dependent cysteine proteases such as calpain-2, which is induced by high levels of intracellular calcium (Liu et al., 2014; Kobeissy et al., 2015). The activation of calpain-2 is associated with numerous downstream consequences, including the cleavage of cytoskeletal proteins into stable proteolytic fragments that can be captured as fluid-based biomarkers (Saatman et al., 2010). Activated calpains can aberrantly cleave several proteins that are critical for the integrity of neurons, such as spectrin, tubulin, tau, microtubule-associated protein, and neurofilaments (Wang and Po-Wai, 1994). Calpain-2 activation might exacerbate tau pathology as TBI mouse models have revealed calpain-2 activation resulted in increased tyrosine phosphorylation of kinase c-Abl at Tyr245, resulting in increased kinase activity and phosphorylation of tau at Tyr394 (Wang et al., 2017). Likewise, RNA-sequencing analyses in postmortem human studies have yielded evidence of a downregulation of PPP3CA, which encodes for calcium-dependent calmodulin-stimulated protein phosphatase, in patients with CTE compared to healthy subjects, and these patterns were inversely correlated with phosphorylation of tau at Ser202/Thr205 (Seo et al., 2017).

Calpains can alter synaptic plasticity by degrading membrane proteins such as glutamate receptors and transporters and can also modulate the function of protein kinases and phosphates. Calpain activation might play an important role in pathological cascades such as diffuse axonal injury, as calpain-mediated proteolysis of spectrin has been detected acutely after injury. Assessment of calpain-mediated N-terminal fragment of spectrin in fluid-based measures such as blood were found to be acutely elevated after injury and predicted the long-term functional effects of the injury in patients with TBI, including professional athletes suffering from concussions (Siman et al., 2013, 2015). Recent studies have revealed that calpain-2 conditional knockout mice are protected against the pathological consequences of repeated mild TBI and that genetic deletion or pharmaceutical inhibition of calpain-2 prevented alterations in the subcellular localization of TDP-43.

Elevation in intracellular calcium in TBI is also associated with activation of caspases, another family of cysteine proteases. Caspase activation following trauma can lead to the proteolytic processing of several proteins that could result in the onset of apoptotic cascades and associated downstream pathological consequences. Sufficient buildup of intracellular calcium also results in the activation of endonucleases following TBI, which can induce DNA damage by altering heterochromatin configurations within the nucleus (Trump and Berezesky, 1995; McConkey and Orrenius, 1996). Furthermore, injury results in the activation of phospholipases, such as PLC and cPLA_2_ that leads to the generation of arachidonic acid, which in turn can increase membrane permeability and elevate levels of reactive oxygen species (ROS). This is turn can lead to cellular damage by peroxidation of lipid membranes, DNA and various signaling proteins (Dhillon et al., 1995, 1999). Following brain injury, results have suggested an increase in expression of DAG and protein kinase C (PKC), key drivers of secondary calcium signaling, which can directly interact with ROCs and VGCCs to further amplify calcium influx into the intracellular space within neurons. Concomitant data have purported that signaling via G protein-coupled receptors that increase Ca^2+^ by IP3 generation appears to be dysregulated after TBI *in vivo* (Delahunty et al., 1995) and with *in vitro* studies using mechanical injury paradigms (Rzigalinski et al., 1998; Weber et al., 1999). Similarly, calmodulin-dependent protein kinase II (CaMKII) expression levels are increased following TBI, which in turn, can alter the permeability of glutamate receptors, alpha-amino-3-hydroxy-5-methyl-4-isoxazole propionic acid receptors (AMPARs), within the synaptic active zone. The multiple mechanisms that exacerbate calcium dysfunction within neurons in TBI lead to impairments in synaptic plasticity as indicated by various studies showing deficits in long term potentiation (LTP) in TBI mouse models (Sick et al., 1998; Albensi et al., 2000; Sanders et al., 2000; Albensi, 2001; Schwarzbach et al., 2006). These studies highlight how calcium signaling is perturbed under conditions of TBI to impair physiological properties of neurons.

### 6.3 Mitochondrial dysfunction and oxidative stress

A key driver of downstream injury cascades in TBI involves mitochondrial impairments and the generation of oxidative stress (Hakiminia et al., 2022). This is mediated, at least in part, by exacerbated intracellular calcium signaling via TBI-induced events, but could be by intrinsic abnormalities in mitochondria. For example, the structural and functional integrity of mitochondria are compromised in TBI, as indicated by disruptions in the electron transfer chain and mitochondrial membrane potential. Mitochondrial dynamics involving fission and fusion events are abrogated in TBI, resulting in a decrease in mitochondrial size and elevated dynamin-related protein 1 (Drp1) localization to mitochondria (Fischer et al., 2016). This in turn, causes mitochondrial depolarization and depleted ATP production, compromising mitochondrial function within neurons (Ladak et al., 2019). Suppression of Drp1 with mitochondrial division inhibitor-1, rescued the decrement in mitochondrial length, thereby preventing hippocampal neuron apoptosis, and enhancing cognition in TBI (Fischer et al., 2016). Excessive intracellular calcium results in calcium overload of mitochondria, subsequently resulting in the generation of reactive oxygen species (ROS).

Reactive oxygen species (ROS) can induce a myriad of pathophysiological events, including the synthesis and release of inflammatory cytokines, compromising the integrity of the blood-brain barrier, and with more pronounced changes causing widespread pathological changes, including cerebral perfusion, ischemia and edema (Mendes Arent et al., 2014; Najem et al., 2018). The activation of these deleterious pathways can invoke apoptotic and necroptosis pathways. For example, TBI is associated with the upregulation of necroptosis pathways, such as receptor-interacting protein kinase 3 (RIPK3) and mixed lineage kinase domain-like protein (MLKL) (Liu et al., 2018; Ni et al., 2019). Inhibition of necroptosis pathways with necrostatin-1 attenuated tissue damage and enhanced functional outcomes in the controlled cortical impact TBI model deficient in TNF-α and Fas (You et al., 2008). Histopathological analysis in postmortem human tissue in TBI patients in the peri-ischemic zone (PIZ) of traumatic cerebral contusions has further defined increases in Bax, Bcl-2 and caspase-3 (Nathoo et al., 2004). Furthermore, postmortem evaluations in the pericontusional zone of TBI patients revealed an upregulation of apoptosis with TUNEL-positive staining, and these alterations were associated with poorer prognosis after TBI (Miñambres et al., 2008). The destabilization of mitochondria and generation of oxidative stress could ultimately also promote disruptions in the autophagy-lysosomal pathways (Clark et al., 2008; Lai et al., 2008).

### 6.4 Activation of inflammatory pathways

The inflammatory mechanisms that result from acute and chronic brain injury is mediated by both innate and adaptive immune system responses. Although the temporal sequence and the involvement of specific components of the immune system are currently under investigation, several preclinical studies in rodent TBI models have illuminated the role of specific cell-types in the cascade. Following acute injury, cytokines, chemokines, and damage-associated molecular patterns (DAMPs) are thought to involve recruitment of various key cell-types. This directly involves the recruitment of neutrophils that constrain the site of injury and facilitate the elimination of damaged cells and cellular debris. This is associated with the recruitment of microglia and astrocytes acutely to perform neuroprotective roles, oftentimes within 3–5 days post-injury. At later time points, T and B cells are involved to partake in restorative roles (McKee and Lukens, 2016).

Damage-associated molecular patterns (DAMPs) are released under conditions of cellular stress to initiate an inflammatory cascade. This involves the activation of nuclear factor kappa-light-chain-enhancer of activated B cells (NF-κB) inducing kinase (Lee et al., 2013), in conjunction with key proinflammatory cytokines TNF-α and IL-6 (Hang et al., 2005). Studies in rat models of TBI and in human subjects have shown high expression of High Mobility Group Box 1 protein (HMGB1), a protein that binds to TLR4 and propels activation of inflammatory pathways (Gao et al., 2012). Intriguingly, anti-HMGB1 targeting therapies are being pursued as a mechanism to attenuate inflammatory pathways and ameliorate the breakdown of the blood-brain barrier (BBB) following injury (Okuma et al., 2012). This is accompanied by the upregulation of pathways that mediate antigen presentation [e.g., major histocompatibility complex (MHC) II], phagocytosis related pathways such as C3, C4 and FCGR4, CD86 (Lagraoui et al., 2012), pathways that promote pro-inflammatory cytokines (e.g., IL-10, IL-6, IL-2, IFN-γ, IL-1β) and chemokines expression (e.g., CXCL4, CCL4, CCL2). For example, studies in TBI have shown IL-1β is upregulated in subjects with serious injuries for 24 h, and correlated with decreased long-term functional outcomes (Hayakata et al., 2004). Studies in rodents have shown that antagonism of IL-1β results in a reduction in anatomical and functional consequences of neuroinflammation (Jones et al., 2005). Similar studies conducted for IL-18 have identified this cytokine as being expressed early in TBI and administration of a specific inhibitor for IL-18 in mice showed a significantly improved neurological recovery by 7 days, accompanied by attenuated intracerebral IL-18 levels (Yatsiv et al., 2002). Findings have shown that early administration of anti-inflammatory cytokines, e.g., IL-10 and TGF-β, can ameliorate the neurological effects in different TBI models (Knoblach and Faden, 1998; Maas et al., 2008). Furthermore, a critical driver of inflammation involves inflammasome activation in TBI (Liu et al., 2013), and studies in human subjects have shown that inflammasome components are detected in cerebrospinal fluid (CSF) are associated with worse clinical outcomes (Adamczak et al., 2012).

### 6.5 Neurodegeneration involving diffuse axonal injury (DAI)

Postmortem studies in human subjects have revealed diffuse axonal injury (DAI) as a common neuropathological feature of exacerbated TBI, including Wallerian-like degeneration following injury with white matter atrophy. In recent studies, human and animal studies have also linked DAI as a key pathological substrate of concussion (Smith and Meaney, 2000; Meaney and Smith, 2011; Johnson et al., 2013; Smith et al., 2013). TBI has been shown to generate toxic tau epitopes and Aβ in degenerating axons, although the precise molecular mechanisms that lead to these interactive phenomena have yet to be elucidated (Goldstein et al., 2012; Johnson et al., 2012; Tagge et al., 2018). Physical injury has been hypothesized to create shearing forces that abrogate the function of the cytoskeleton network, eventually inducing microstructural breaks that reduce the efficacy of axonal transport. This could lead to the mislocalization of key components of Aβ generation in the axonal swellings, including amyloid precursor protein (APP) and enzymes that cleave APP such as beta secretase 1 and presenilin 1. This is believed to lead to the intracellular production of Aβ that is released to produce extracellular Aβ plaques. Similarly, mechanical shearing forces can lead to detachment of pathogenic tau from microtubules which mediates hyperphosphorylation and aggregation of *cis* phosphorylated tau.

### 6.6 Glymphatic system disruption

The brain clearance involves the removal of waste via multiple, overlapping networks which include active and passive transport via the blood brain barrier, diffusion and the glymphatic system. The glymphatic system is comprised of subarachnoid cerebrospinal fluid flowing into the brain adjacent to arteries, mixing with the brain interstitial fluid (ISF) with subsequent outflow along cerebral venous system (Butler et al., 2023). Compromised glymphatic system clearance has been associated with toxic protein accumulation, including Aβ and tau, in turn potentially leading to development of Alzheimer’s disease (Tarasoff-Conway et al., 2015). The clearance of toxins and waste is particularly compromised in TBI, in which various neuronal debris including Aβ and tau have to be cleared. Such disruption has formed one of the correlational hypotheses linking TBI and increased risk of Alzheimer’s disease and various neurodegenerative disorders (Johnson et al., 2010). A novel imaging technique, referred to as diffusion tensor imaging along perivascular spaces (DTI-ALPS), has been utilized to assess glymphatic system disruption post TBI. DTI-ALPS based findings post subacute TBI support a correlation between disease severity based on blood levels of neurofilament light protein (NfL), a highly sensitive TBI biomarker, and glymphatic system disruption (Butler et al., 2023). The role of aquaporin 4 (AQP4) in immediate brain damage within 72 h of injury has been examined and shown to be altered in rodent TBI studies, in turn suggesting its role in ISF transport (Liu et al., 2020). Recent evidence also shows the glymphatic exchange to be significantly enhanced during sleep, and suppressed during normal wakefulness hours (Xie et al., 2013). These findings support a potential bidirectional relationship between TBI, sleep disruption and glymphatic clearance (Piantino et al., 2019). The emerging studies linking TBI, glymphatic disruption, sleep and risk of neurodegenerative disorders support a complex dynamic association between specific trauma induced physiological perturbation and chronic neuropathological changes. Hence the glymphatic system integrity and functioning post-injury would likely serve as a robust biomarker of TBI severity and a predictive marker for potential neurodegenerative sequelae (Peters and Lyketsos, 2023).

## 7 Conclusion and perspective

As per Center of Disease Control and Prevention (CDC), TBI resulted in 223,000 hospitalizations and 64,300 deaths in 2020 (Peterson et al., 2022). As TBI remains to be a major cause of morbidity and mortality worldwide, there is increasing demand on enhancing our collective understanding of the underlying pathophysiology and chronic neuropsychiatric sequelae post TBI. Given the inherent complexity and heterogeneity of TBI, the animal models of TBI have created an essential biological substrate in which various biomechanical parameters, including severity of injury and timeline of the injury and post-injury analysis, could be controlled, manipulated and comprehensively studied. Moreover, the animal models of TBI have created the opportunity to examine various disease modifying therapeutic agents with significant translational value. As there are currently no US Food and Drug Administration (FDA)-approved and specific regimen for TBI induced neurocognitive and neuropsychiatric sequelae (Fesharaki-Zadeh, 2023), there is an ever-increasing necessity for evolving the current TBI models with the ultimate goal of the closest possible and accurate disease simulation. The development of recent TBI models, including CHIMERA, is one such pertinent case. The evolving field of biomarker studies of TBI in conjunction with expanding studies of animal models of TBI, would provide a necessary complementary development that would provide the essential translational tool for future novel therapeutic modalities.

## Author contributions

AF-Z: Conceptualization, Methodology, Resources, Writing – original draft, Writing – review & editing. DD: Conceptualization, Writing – original draft, Writing – review & editing.
